# Sphingosine 1-Phosphate Receptor 1 as a Useful Target for Treatment of Multiple Sclerosis

**DOI:** 10.3390/ph5050514

**Published:** 2012-05-18

**Authors:** Kenji Chiba, Kunitomo Adachi

**Affiliations:** Research Division, Mitsubishi Tanabe Pharma Corporation, 1000, Kamoshida-cho, Aoba-ku, Yokohama, Kanagawa 227-0033, Japan; Email: Adachi.Kunitomo@mc.mt-pharma.co.jp (K.A.)

**Keywords:** sphingosine 1-phasphate (S1P), S1P receptor 1 (S1P_1_), fingolimod (FTY720), lymphocyte egress, immunomodulator, experimental autoimmune encephalomyelitis (EAE), multiple sclerosis (MS), therapy

## Abstract

Sphingosine 1-phosphate (S1P), a lysophospholipid mediator, is generated from sphingosine by sphingosine kinases and binds five known cell surface receptors. S1P receptor 1 (S1P_1_) plays an essential role in lymphocyte egress from secondary lymphoid organs (SLO), as evinced by the inability of lymphocytes to exit from the SLO in mice lacking lymphocytic S1P_1_. Fingolimod hydrochloride (FTY720) is a first-in-class, orally active, S1P receptor modulator with a structure closely related to sphingosine. FTY720 was first synthesized by chemical modification of a natural product, myriocin. FTY720 is effectively converted to an active metabolite, FTY720 phosphate (FTY720-P) by sphingosine kinases. FTY720-P shows high affinity to 4 of the S1P receptors (S1P_1_, S1P_3_, S1P_4_, and S1P_5_). In particular, FTY720-P strongly induces internalization and degradation of S1P_1_, inhibits S1P responsiveness of lymphocytes in the SLO, and acts as a functional antagonist at lymphocytic S1P_1_. Consequently, FTY720 inhibits S1P_1_-dependent lymphocyte egress from the SLO to decrease circulation of lymphocytes including autoreactive Th17 cells and is highly effective in experimental autoimmune encephalomyelitis (EAE), an animal model of multiple sclerosis (MS). Because FTY720 shows a superior efficacy in relapsing remitting MS patients compared to intramuscular interferon-β-1a (Avonex^®^), S1P_1_ is presumed to be a useful target for the therapy of MS.

## 1. Introduction

A potent immunosuppressive natural product, (2*S*,3*R*,4*R*)-(*E*)-2-amino-3,4-dihydroxy-2-(hydroxy-methyl)-14-oxoeicos-6-enoic acid, (ISP-I = myriocin = thermozymocidin) was isolated from culture broths of *Isaria sinclairii*, a fungus which attacks insects [1]. Extensive modifications of ISP-I were conducted and simplification of the structure of ISP-I including removal of the side chain functionalities as well as elimination of chiral centers led to a novel compound, 2-amino-2-[2-(4-octylphenyl)ethyl]propane-1,3-diol (FTY720, fingolimod hydrochloride) with more potent immunosuppressive activity and less toxicity compared with ISP-I [2,3]. Although ISP-I inhibits serine-palmitoyl-transferase, the first enzyme in sphingolipid biosynthesis, FTY720 showed no effect on this enzyme activity, suggesting that FTY720 possesses a new mechanism of action distinct from ISP-I [4].

FTY720 at an oral dose of 0.1 mg/kg or higher significantly prolongs allograft survival in various experimental allotransplantation models and autoimmune disease models [5,6,7,8,9]. Unlike calcineurin inhibitors, FTY720 does not impair lymphocyte function including cytokine production by helper T cells [10,11]. A striking feature of FTY720 is the induction of a marked decrease in the number of peripheral blood lymphocytes (T cells and B cells) at doses that show immunosuppressive effects [5,10]. The reduction of peripheral blood lymphocytes by FTY720 is predominantly caused by sequestration of circulating mature lymphocytes into the secondary lymphoid organs (SLO) and thereby decreasing T cell infiltration into inflammatory sites [10,11,12].

Circulation of mature lymphocytes among the blood, lymph, and SLO plays a central role in the establishment of the immune response to foreign antigens. Homing of lymphocyte from blood into the SLO beyond high endothelial venules is highly dependent on the interaction between the chemokines and their receptors on lymphocytes [13]. On the other hand, it has been clarified that a lysophospholipid mediator, sphingosine 1-phosphate (S1P), and its receptor type 1 (S1P_1_) play an important role in lymphocyte egress from the SLO and thymus throughout the analyses of the mechanism of action of FTY720 [8,13,14].

FTY720 is effectively converted to an active metabolite, FTY720 phosphate (FTY720-P) by sphingosine kinases [15,16]. FTY720-P induced internalization and degradation of S1P_1_, almost completely inhibits S1P responsiveness of lymphocytes in the SLO, and acts as a functional antagonist at lymphocytic S1P_1_ [8,17,18,19]. Consequently, FTY720 inhibits S1P_1_-dependent lymphocyte egress from the SLO to decrease the number of peripheral blood lymphocytes. This paper summarizes the current understanding of FTY720, a functional antagonist at S1P_1_, and discusses about the feasibility of S1P_1_ as a useful target for treatment of multiple sclerosis (MS), an autoimmune diseases in the central nervous system (CNS).

## 2. Discovery of FTY720

**Figure 1:**
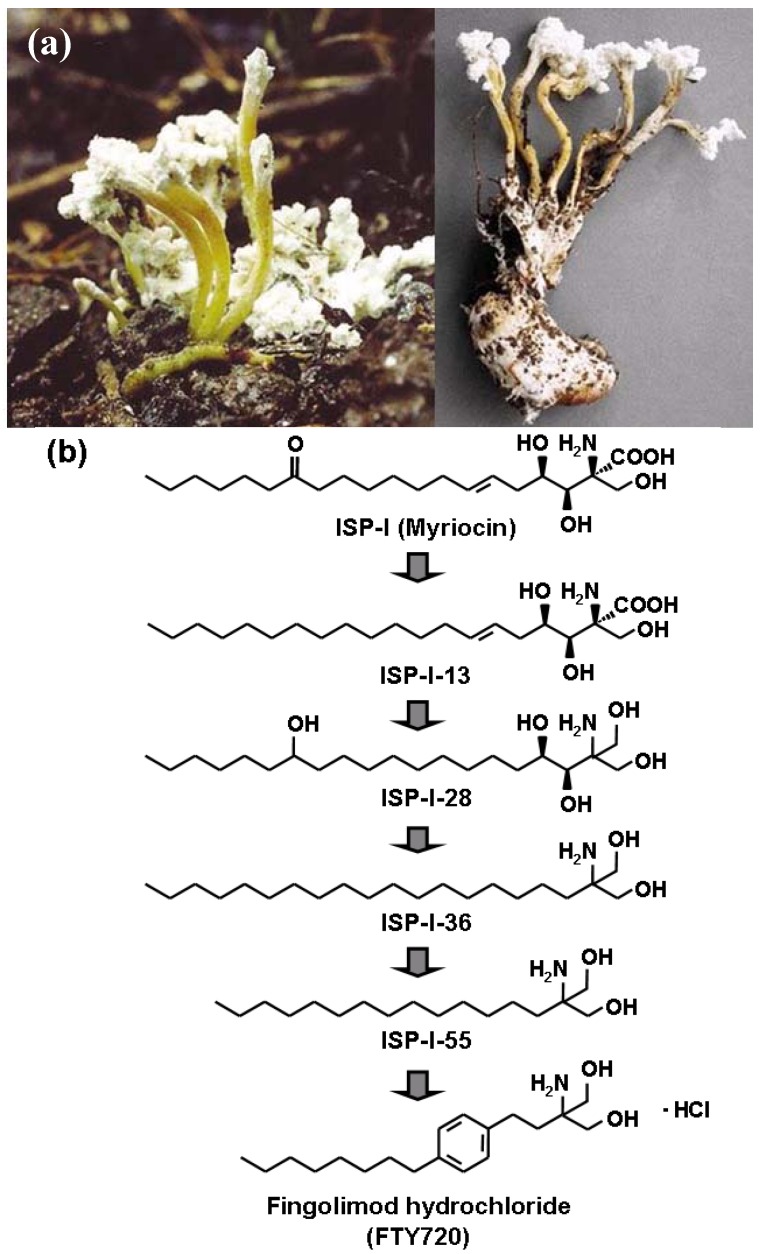
(**a**) *Isaria sinclairii* [21] (**b**) Discovery of fingolimod hydrochloride (FTY720) by lead optimization based on structure simplification starting from ISP-I.

A potent immunosuppressive natural product, ISP-I was isolated from a culture broth of *Isaria sinclairii*, an entomopathogenic fungus that is an “eternal youth” nostrum in traditional Chinese medicine [1,20] (Figure 1a). ISP-I is a rather complicated amino acid with three successive asymmetric centers and some functionalities (Figure 1b). ISP-I at nano-molar concentrations strongly inhibited the proliferation of T cells in mouse allogeneic mixed lymphocyte reaction (MLR) *in vitro* [1]. Moreover, ISP-I (0.3 mg/kg, intraperitoneally) significantly prolonged rat skin allograft survival; however higher dose of ISP-I induced strong toxicity *in vivo* [1]. ISP-I-13 (deoxomyriocin) (Figure 1b) showed 3-fold more potent activity than ISP-I *in vitro* but neither of toxicity nor solubility was improved [20].

Based on these results of ISP-I and ISP-I-13, we performed lead optimization using both allogeneic MLR assay *in vitro* and rat skin allograft *in vivo* as screens [1,2,3,4,20,22]. The structure-activity relationship studies on these analogues including ISP-I and their semi-synthetic derivatives revealed that both the functionalities (hydroxyl at position 4, olefin at position 6, and carbonyl at position 14) and the absolute configuration at the carbon bearing the 3-hydroxy group are less important for its activity than the other functionalities [2,3]. Therefore, simplification of the structure of ISP-I was conducted to reduce its toxicity and to improve its physicochemical properties.

The simplification was focused on removal of the side chain functionalities and elimination of asymmetric centers. During the course of the process, ISP-I-28 (Figure 1a) with a hydroxymethyl group instead of the carboxylic acid of ISP-I was found to be more active *in vivo* but also to be much less toxic than ISP-I [2,3]. Further simplification led to much more simplified compounds having a 2-alkyl-2-aminopropane-1,3-diol framework such as ISP-I-36 and ISP-I-55 [5,6,7,8,9,10] (Figure 1a). The latter was further modified by introducing a phenylene moiety in a proper position within the side chain and FTY720 was discovered [2,3,4,22] (Figure 1a). FTY720 shows more potent immunosuppressive activity than ISP-I-28, ISP-36, or ISP-I-55 *in vivo*. The drastic structure simplification of ISP-I was effective in improving *in vivo* immunosuppressive activity, toxicity, and physicochemical properties, leading to the development of FTY720 [2,3,4,22,23].

ISP-I was reported to inhibit interleukin (IL) 2-dependent proliferation of mouse cytotoxic T cell line (CTLL-2 cells) by inhibiting serine-palmitoyl-transferase involved in sphingolipid biosynthesis [4]. It was surprising that neither FTY720 nor ISP-I-55 displayed inhibitory activity for this enzyme [4], suggesting the serendipitous discovery of a new mechanism of action during the optimization process from ISP-I to FTY720.

## 3. Mechanism of Action of FTY720

### 3.1. FTY720 Sequesters Circulating Lymphocytes into the SLO

FTY720 at an oral dose of 0.1 mg/kg or higher significantly prolongs allograft survival and shows a synergistic effect in combination with calcineurin inhibitors (cyclosporine A and tacrolimus) in experimental skin, cardiac and renal allotransplantation models [5,6,7,8,10,12,17,24,25]. Moreover, oral administration of FTY720 is highly effective in various autoimmune disease models including experimental autoimmune encephalomyelitis (EAE), adjuvant- or collagen-induced arthritis in rats and mice, and lupus nephritis in MRL/*lpr* mice [8,9,17,26]. Unlike calcineurin inhibitors, FTY720 does not impair lymphocyte function including T cell activation and production of IL-2 and interferon (IFN)-γ by type 1 helper T cells (Th1 cells) [5,10,11].

A striking feature of FTY720 is the induction of a marked decrease in the number of peripheral blood lymphocytes at doses that show immunosuppressive effects [5,10]. When FTY720 at an oral dose of 0.1 mg/kg or higher is given to rats or mice, the number of lymphocytes is decreased markedly in the peripheral blood and thoracic duct lymph whereas that in the SLO is increased significantly [10]. Intravenous transfusion of fluorescein-labeled lymphocytes into rats revealed that the labeled lymphocytes are accumulated in the SLO by FTY720 administration [10]. These data strongly suggest that FTY720 induces sequestration of circulating mature lymphocytes into the SLO and decreases the number of lymphocytes in peripheral blood and lymph. Accordingly, the sequestration of circulating mature lymphocytes is presumed to be the main mechanism of immunosuppressive activity of FTY720.

### 3.2. Role of S1P and S1P_1_ Receptors in Lymphocyte Egress from the SLO

**Figure 2:**
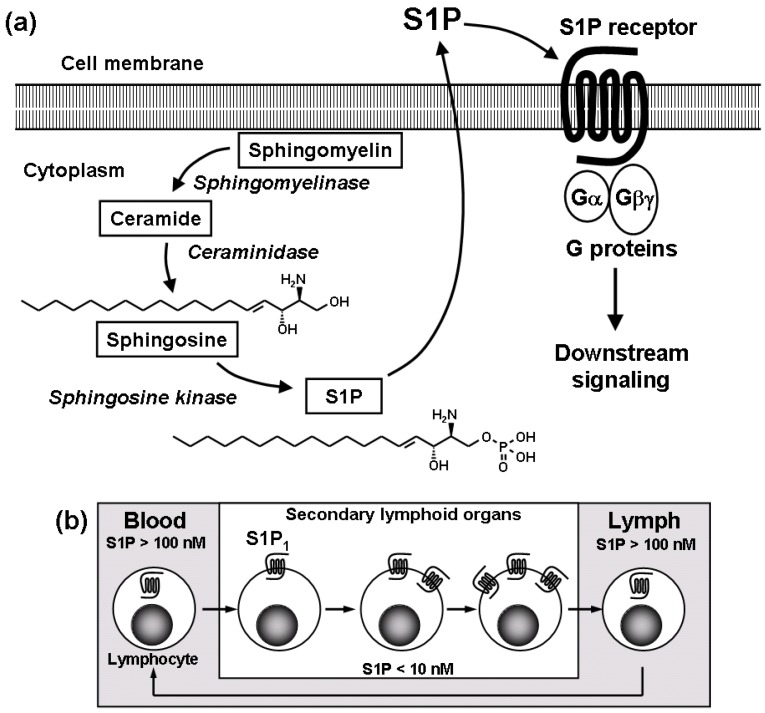
S1P_1_ plays an essential role in lymphocyte egress from the SLO. (**a**) S1P is generated from sphingosine by sphingosine kinases and binds to S1P receptors; (**b**) Lymphocytic S1P_1_ is down-regulated in the blood, up-regulated in the SLO, and down-regulated again in the lymph [23].

Throughout the analyses of the molecular mechanism of FTY720, it has been emphasized that S1P and S1P_1_ play an important role in lymphocyte egress from the SLO and thymus [8,13,14,17]. S1P, a pleiotropic lysophospholipid mediator, is generated primarily by the phosphorylation of intracellular sphingosine by sphingosine kinases (Figure 2a).

S1P stimulates multiple signaling pathways resulting in calcium mobilization from intracellular stores, polymerization of actin, chemotaxis/migration, and escape from apoptosis. Significant amounts of S1P (100 to 400 nM) are found in blood and lymph whereas the S1P levels in the SLO are relatively low (<10 nM), indicating a concentration gradient of S1P existing between blood-lymph and SLO (Figure 2b) [13,14,27]. Plasma S1P is tightly associated with albumin and lipoproteins, particularly high-density lipoprotein and the major source of plasma S1P is red blood cells and platelets [28]. Excessive production of S1P can be induced at inflammatory sites as a result of cell activation by pro-inflammatory cytokines. The S1P gradient between blood-lymph and SLO, as well as over production of S1P at inflammatory sites, appears to play an important role in regulation of lymphocyte trafficking.

S1P binds with subnano to nano-molar affinities to five related G-protein-coupled receptors, termed S1P_1–5_ [29,30]. S1P_1_, S1P_2_, and S1P_3_ receptors are widely expressed in the immune, cardiovascular, and central nervous systems. S1P_4_ is selectively expressed in lymphoid tissues and lung whereas S1P_5_ is expressed in spleen and white matter tracts of the central nervous systems. The expression of S1P_1_ mRNA in CD4 T cells is markedly higher than the other S1P receptors, suggesting that S1P_1_ is the dominant receptor on lymphocytes.

It has been reported that S1P_1_ is essential for lymphocyte recirculation and that S1P_1_ regulates lymphocyte egress from the SLO [13,14,17]. In mice whose hematopoietic cells lack a single S1P receptor, S1P_1_, there are no T cells in the periphery because mature T cells are unable to exit the SLO [14]. Moreover, S1P at concentrations of 10 to 100 nM induces migration of lymph node CD4 T cells in mice [8,14]. S1P-induced migration was extremely low level in lymphocytes from S1P_1_-deficient mice, suggesting S1P induces lymphocyte migration via lymphocytic S1P_1_ [14]. S1P_1_-dependent migratory responsiveness is suggested to be up-regulated in lymphocytes before exit from the SLO, whereas S1P_1_ is down-regulated during peripheral lymphocyte activation, and this is associated with retention of lymphocytes in the SLO [27]. Because S1P_1_ surface expression on lymphocytes is highly dependent on the extracellular concentration of S1P, S1P_1_ on lymphocytes is down-regulated in the blood, up-regulated in the SLO and down-regulated again in the lymph (Figure 2b). Consequently, it is proposed that cyclical modulation of S1P_1_ surface expression on circulating lymphocytes by S1P contributes to establishing their transit time in SLO [27].

### 3.3. FTY720 Acts as a Functional Antagonist at S1P_1_ Receptor

**Figure 3:**
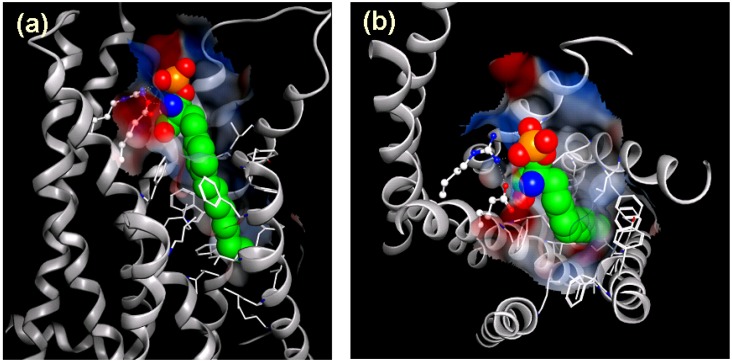
Docking model of S1P_1_ with the (*S*)-enantiomer of FTY720-P. A model of S1P_1_ was constructed by the homology model protocol in Discovery Studio 1.7 (Accelrys Software Inc.). (*S*)-FTY720-P is shown in a sphere model, with carbon in green, oxygen in red, nitrogen in blue, and phosphorus in orange. (**a**) side view, (**b**) view from upper side.

By reverse pharmacological approaches to clarify the mechanism of action of FTY720, it has been demonstrated that like sphingosine, FTY720 is a substrate for sphingosine kinases and that a phosphorylated form of FTY720 (FTY720-P) binds to four types of S1P receptors (S1P_1_, S1P_3_, S1P_4_, and S1P_5_) but not S1P_2_, and acts as a high affinity agonist at these receptors [15,31]. After oral or intravenous FTY720 administration, the plasma concentration of FTY720-P was 2 to 6 times higher than FTY720 [15]. We have confirmed that only the (*S*)-enantiomer of FTY720-P can bind S1P_1_ S1P_3_, S1P_4_, and S1P_5_ (but not S1P_2_) at nano-molar concentrations, whereas FTY720 up to 10,000 nM does not bind S1P receptors [32]. A binding model of S1P_1_ with the (*S*)-enatiomer of FTY720-P is illustrated in Figure 3. A model of S1P_1_ was constructed by the homology model protocol in Discovery Studio 1.7 (Accelrys Software Inc.) based on the bovine rhodopsin crystal structure (PDB code 1F88) as a template.

**Figure 4:**
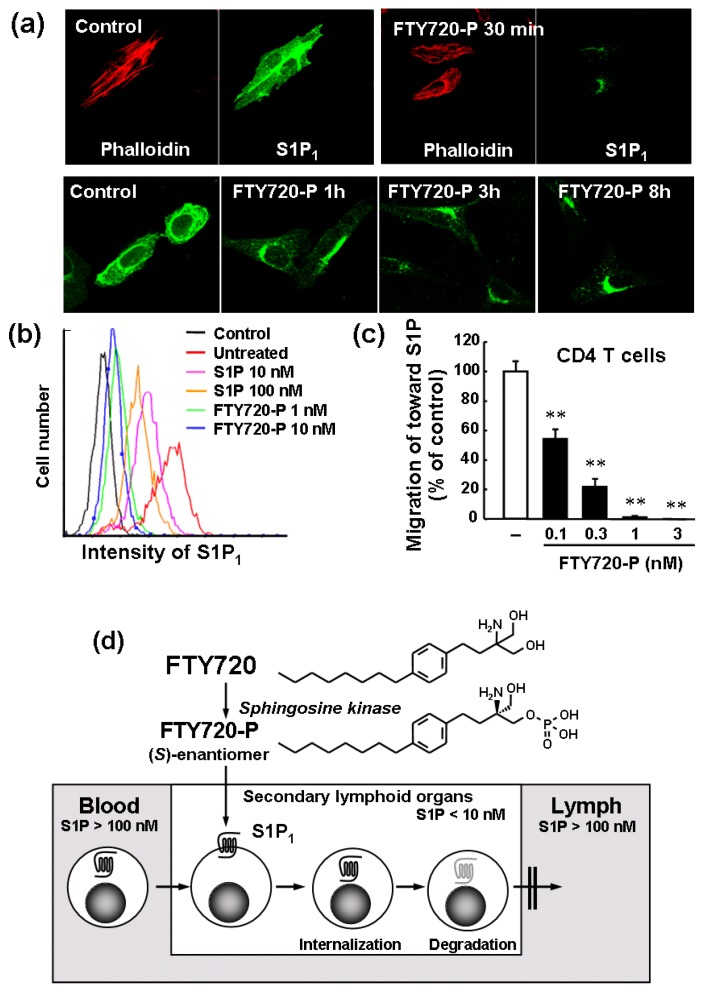
FTY720-P acts as a functional antagonist at lymphocytic S1P_1_ and inhibits lymphocyte egress from the SLO [8,17,18,23]; (**a**) Confocal microscopy of human S1P_1_-expressing CHO cells treated with FTY720-P (100 nM) [8]; (**b**) Human S1P_1_-expressing CHO cells were stained with FITC-conjugated anti-human S1P_1_ monoclonal antibody (mAb) and expression of S1P_1_ on cell surface was analyzed by flow cytometry [17]; (**c**) Pretreatment with FTY720-P inhibits migration of mouse CD4 T cells toward 10 nM S1P [18]; (**d**) FTY720-P converted from FTY720 inhibits S1P_1_-dependent lymphocyte egress from the SLO by internalization and degradation of lymphocytic S1P_1_ [23].

FTY720-P shows agonist activity for S1P_1_ at nano-molar concentrations using extracellular signal regulated kinase 1/2 (ERK1/2) phosphorylation assay and subsequently induces long-term internalization and degradation of S1P_1_ in Chinese hamster ovary (CHO) cells stably expressing human S1P_1_ (Figure 4a,b) [8,17,18,33].

Consequently, FTY720 treatment down-regulates S1P_1_, creating a temporary pharmacological S1P_1_-null state in lymphocytes, providing an explanation for the mechanism of FTY720-induced lymphocyte sequestration. The down-regulation of S1P_1_ by FTY720-P appears to be maintained longer than that by S1P because FTY720-P but not S1P induces degradation of internalized S1P_1_. The pretreatment with FTY720-P effectively inhibits the migration of CD4 T cells toward S1P (Figure 4c) [8,18]. Based on these results, it is highly likely that FTY720-P converted from FTY720 acts as a functional antagonist at S1P_1_ by internalization and degradation of this receptor, reduces S1P responsiveness of lymphocytes in the SLO, and inhibits S1P_1_-dependent lymphocyte egress from the SLO (Figure 4d).

## 4. Effects of FTY720 on Experimental Autoimmune Encephalomyelitis

**Figure 5:**
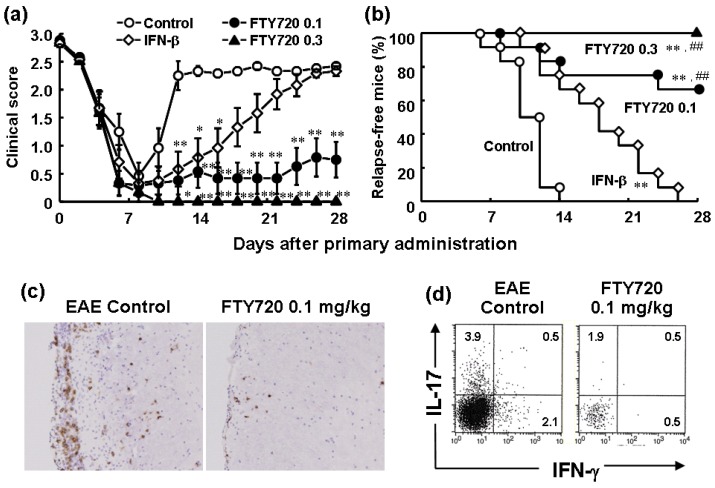
Therapeutic effects of FTY720 and rm-IFN-β on EAE induced by PLP in SJL/J mice [38]. SJL/J mice were immunized with PLP_139–151_ (50 μg/mouse) and Freund’s complete adjuvant. EAE-developed mice were divided into 5 groups on day 15 after immunization and were given FTY720 (0.1 and 0.3 mg/kg orally, everyday) or rm-IFN-β (10,000 IU/mouse subcutaneously, every other day) for 4 weeks. (**a**) Clinical scores are expressed as the mean ± S.E.M. of 12 mice. Statistical differences were calculated by Steel’s test (* *p* < 0.05, ** *p* < 0.01 *versus* control); (**b**) Mice remaining relapse-free. Statistical differences were calculated by generalized Wilcoxon test (** *p* < 0.01 *versus* control, ## *p* < 0.01 *versus* IFN-β); (**c**) Immunohistochemical staining of the spinal cords with anti-mouse CD4 mAb; (**d**) Intracellular cytokine staining of spinal cord lymphocytes in EAE mice was performed by using anti-CD4, anti-IL-17, and anti-IFN-γ mAbs.

Oral administration of FTY720 is highly effective in experimental autoimmune encephalomyelitis (EAE), a CD4 T cell-dependent model for multiple sclerosis (MS) [26,34,35,36,37,38]. The development of EAE induced by myelin proteolipid protein (PLP) in SJL/J mice is almost completely prevented and infiltration of CD4 T cells into the spinal cord is decreased by prophylactic treatment with FTY720 or FTY720-P [26,34]. When FTY720 (0.1 and 0.3 mg/kg orally) is given after establishment of EAE induced by PLP in SJL/J mice, the relapse of EAE is significantly inhibited as compared with recombinant mouse IFN-β (10,000 international units (IU)/mouse subcutaneously) (Figure 5a,b), and the infiltration of CD4 T cells are markedly decreased in the spinal cords of EAE mice (Figure 5c) [26,38]. Similar therapeutic effects by FTY720 are obtained in EAE induced by myelin oligodendrocyte glycoprotein (MOG) in C57BL/6 mice [26,38].

It has been reported that infiltration of encephalitogenic CD4 T cells, particularly IL-17-expressing helper T cells (Th17 cells), into the CNS plays a critical role in the development and progression of EAE in mice [39,40,41]. Oral administration of FTY720 at 0.1 mg/kg or higher significantly inhibits the development of EAE (Figure 5a,b) and markedly reduces the frequency of Th17 cells in the spinal cords of EAE mice (Figure 5d) [38]. On the contrary, the frequency of Th17 cells in draining inguinal lymph nodes is significantly increased by FTY720, suggesting sequestration of myelin antigen-specific Th17 cells into the draining lymph nodes [38]. Moreover, Th17 cells can migrate toward 10 nM S1P and the pretreatment with 1 nM FTY720-P almost completely inhibits S1P-induced migration of Th17 cells [42]. On the other hand, FTY720-P up to 100 nM shows no clear effect on generation of Th 17 cells or IL-17 production by them. Consequently, the ameliorating effects of FTY720 on EAE are likely due to reduction of infiltration of encephalitogenic Th17 cells into the CNS.

In EAE induced by myelin basic protein in LEW rats, prophylactic administration of FTY720 (0.1 to 1 mg/kg orally) almost completely prevents the development of EAE symptoms, and therapeutic treatment with FTY720 significantly inhibits the progression of EAE and EAE-associated histological change in the spinal cords [26]. In EAE induced by MOG in DA rats, prophylactic therapy of FTY720 protects against the emergence of EAE symptoms, neuropathology, and disturbances to visual and somatosensory evoked potentials [35,37]. Moreover, therapeutic treatment of FTY720 markedly reverses paralysis in established EAE and normalizes the electrophysiological responses with decreased demyelination in the CNS [35].

It has been thought that the efficacy of FTY720 in EAE is partly due to additional direct effects in the CNS because neural cells (neuron, astrocytes, oligodendrocytes, and microglia) constitutively express S1P receptors. Recently, it has been strongly suggested that FTY720-P directly acts as a functional antagonist at S1P_1_ on neural cells, particularly astrocytes [43,44] because astrocytes express S1P_1_ and FTY720 can distribute into the CNS beyond blood brain barrier [37]. EAE was attenuated and FTY720 efficacy was lost in CNS mutants lacking S1P_1_ on glial fibrillary acidic protein-expressing astrocytes but not on neurons, suggesting the loss of S1P_1_ on astrocytes through functional antagonism by FTY720-P as a primary FTY720 mechanism [43,44]. Consequently, it is likely that the therapeutic effects of FTY720 on EAE is likely due to a culmination of mechanisms involving reduction of myelin antigen-specific T cells, neuroprotective influence of FTY720-P in the CNS, and inhibition of inflammatory mediators in the brain.

## 5. Clinical Trails of FTY720 in MS

MS is a common and often disabling autoimmune disease of the CNS. Early active MS lesions are characterized by the presence of infiltrated mononuclear cells around venules and small veins, followed by myelin breakdown and astrogliosis, resulting in irreversible disability. The etiology of MS remains unknown, but is widely considered to involve myelin-specific autoimmune destruction mediated by auto-reactive T cells [45,46]. IFN-β, cyclophosphamide, or glatiramer acetate is used for MS therapy [47,48].

The first clinical evidence that FTY720 has therapeutic benefits in MS was provided in a 6-month, placebo-controlled Phase II trial involving 281 patients with relapsing MS [49]. Patients receiving FTY720 at an oral dose of 1.25 mg or 5.0 mg daily had a significant lower median total number of gadolinium-enhancing lesions (the primary end point) on magnetic resonance imaging (MRI) than those receiving placebo. The annualized relapse rates in groups given 1.25 mg and 5.0 mg of FTY720 were 0.35 and 0.36, respectively and were significantly lower than that in the placebo group (0.77). By extension study for additional 6 months, the number of gadolinium-enhanced lesions and relapse rates remained low in groups given FTY720 and both measures decreased in patients who switched from placebo to FTY720. From these results, it is demonstrated that oral FTY720 reduces the number of lesion detected on MRI and clinical disease activity in relapsing MS patients.

In FTY720-treated MS patients, the number of IL-17-expressing CD4 T cells was reduced by >95% in the peripheral blood suggesting that FTY720 inhibits egress of Th17 cells from the SLO and reduces the infiltration of Th17 cells into the CNS [43,50]. In addition, FTY720 primarily reduced the numbers of CCR7^+^ CD45RA^+^ naïve T cells and CCR7^+^ CD45RA^−^ central memory T cells in the blood in MS patients, because these T cells express the homing receptor CCR7, recirculate through the lymph nodes, and can be sequestered into the lymph nodes by FTY720 [43,50]. In contrast, CCR7^−^ CD45RA^−^ and CCR7^−^ CD45RA^+^ effector memory T cell subsets are not sequestered into the SLO and remain in the blood when FTY720 is administered [43,50]. These results suggest that FTY720 effectively inhibits infiltration of pathogenic Th17 cells into the CNS in MS patients whereas FTY720 does not affect the function of effector memory T cells that play an important role in the prevention of systemic infection.

FTY720 was evaluated in a 24-month, double blind Phase III study (FREEDOMS study), involving 1s272 patients with relapsing remitting MS [51]. The patients were randomized to receive a daily oral dose of FTY720 at 0.5 mg or 1.25 mg, or placebo. The annualized relapse rates in groups given 0.5 mg and 1.25 mg of FTY720 were 0.18 and 0.16, respectively and were significantly lower than that in the placebo group (0.40). FTY720 at 0.5 mg and 1.25 mg significantly reduced the risk of disability progression over 24-month period. The cumulative probability of disability progression confirmed after 3 months was 17.7% with 0.5 mg FTY720, 16.6% with 1.25 mg FTY720, and 24.1% with placebo. FTY720 at 0.5 mg and 1.25 mg showed improved effects compared with placebo with regard to the MRI-related measures (number of new or enlarged lesions on T2-weightend images, gadolinium-enhanced lesions, and brain-volume loss).

FTY720 was also evaluated in a 12-month, double blind, double dummy Phase III study (TRANSFORMS study) involving 1,292 patients with relapsing remitting MS, comparing FTY720 with IFN-β-1a (Avonex^®^), an established therapy for MS [52]. Patients were randomized to receive a daily dose of 0.5 mg or 1.25 mg FTY720 orally, or a weekly intramuscular injection of IFN-β-1a. The annualized relapse rates in groups given FTY720 0.5 mg and 1.25 mg were 0.16 and 0.20 respectively, and were significantly lower than that in the group receiving IFN-β-1a (0.33).

## 6. Conclusions

FTY720, discovered by chemical modification of ISP-I, has a structure closely related to sphingosine and is phophorylated by sphingosine kinases. Phosphorylated FTY720 [FTY720-P, the (*S*)-enantiomer] acts as a functional antagonist at S1P_1_ on lymphocytes and neural cells. FTY720 showed superior efficacy as compared with in mouse EAE. FTY720 blocks infiltration of myelin-specific Th17 cells into the CNS by inhibiting lymphocyte egress and showed superior efficacy as compared with rm-IFN-β in mouse EAE. Oral FTY720 showed superior efficacy compared with intramuscular IFN-β-1a (Avonex^®^) and placebo with regard to reducing the rate of relapse and the number of inflammatory lesions in the CNS in relapsing remitting MS patients. FTY720 (Gilenya^®^/Imusera^®^) has been approved as a new therapeutic drug for MS in more than 50 countries including US, EU, and Japan. Based on these results, S1P_1_ appears to be a useful target for the treatment of MS and that functional antagonism at S1P_1_ by FTY720 can provide a new approach for MS therapy.
